# Beef production and carcass evaluation in Brazil

**DOI:** 10.1093/af/vfad074

**Published:** 2024-04-16

**Authors:** Cris Luana de Castro Nunes, Sérgio Bertelli Pflanzer, Jonatã Henrique Rezende-de-Souza, Mario Luiz Chizzotti

**Affiliations:** Department of Animal Science, Universidade Federal de Viçosa, Viçosa, Brazil; Department of Food Engineering and Technology, University of Campinas, Campinas, Brazil; Department of Food Engineering and Technology, University of Campinas, Campinas, Brazil; Department of Animal Science, Universidade Federal de Viçosa, Viçosa, Brazil

**Keywords:** beef production, carcass classification, grading, meat quality, market information

**Implications**

- Brazil plays a leading role in the world beef market, possessing one of the largest commercial beef herds and setting records in livestock-derived product export revenues.
- Brazil has no national carcass grading system. Slaughterhouses are responsible for their own metrics to address internal and export market demands.
- The current standard for carcass classification is based on dentition, gender, carcass weight, and subcutaneous/external fat scores.

## Introduction

This paper aims to explore the main characteristics of beef cattle production and carcass evaluation in Brazil. Livestock production in Brazil continues to expand due to the adoption of new specialized technologies (Hötzel and Vandresen, 2022). In this context, Brazil is placed as the main producer of livestock-derived commodities, ranking among the top world producers and exporters of beef, pork, and poultry (USDA, 2023). This review provides an overview of beef production in Brazil, discusses the Brazilian beef sector statistics, the Brazilian system of carcass classification with examples of slaughterhouse beef carcass grading, Brazilians’ attitudes towards meat consumption, and finally recommendations for a new Brazilian beef carcass and grading system.

## Beef Cattle Production Status and Traceability

Beef cattle in Brazil are raised mainly in a tropical climate environment with a wide range of soil conditions of different biomes (Amazon, Caatinga, Pantanal, Atlantic Forest, Pampa, and Cerrado regions). Some biomes such as Pampa and Pantanal provide native grasslands for grazing, providing an excellent environment for the preservation and sustainability of the biomes used for beef cattle production (Lobato et al., 2014). Beef production in Brazil is manifested in two different systems. The first cattle production system is characterized by minimal technological integration and human interference, consequently with very low productivity rates, as exemplified by heifers with poor body condition scores resulting in first breeding occurring at 36 months of age (Lobato et al., 2014). Conversely, the second system involves intensive livestock farming, which incorporates genetic improvement programs by selecting cows and their progenies for better performance, with intensive forage management, sanitary control, and modern management practices (Rosado Júnior and Lobato, 2010). Since Brazil predominantly produces leaner grass-fed beef carcasses, an increase in the number of younger animals on the market can improve the quality of Brazilian beef.

The Brazilian beef cattle production cycle is getting shorter due to the optimization of land use and the adoption of technologies. As identified by Chiari et al., (2021), artificial insemination to improve genetic gain and uniformity in weaning weight, implementation of practices that improve pasture recovery and adoption of more productive forages to maximize pasture occupancy rates, use of feed supplementation, and adoption of good production practices in controlling diseases and parasites are some of the factors contributing to the increase in cattle productivity over the years. Malafaia et al., (2021) projected an increase in meat production per unit area, improvements in meat quality and sustainable systems, adoption of meat origin certifications, fabrication of more value-added meat cuts, and integration of technology to improve process transparency for stakeholders. These megatrends will ensure Brazil’s continued prominence as a major beef exporter by ensuring a robust Brazilian beef cattle supply chain through 2040.

**Figure 1.**
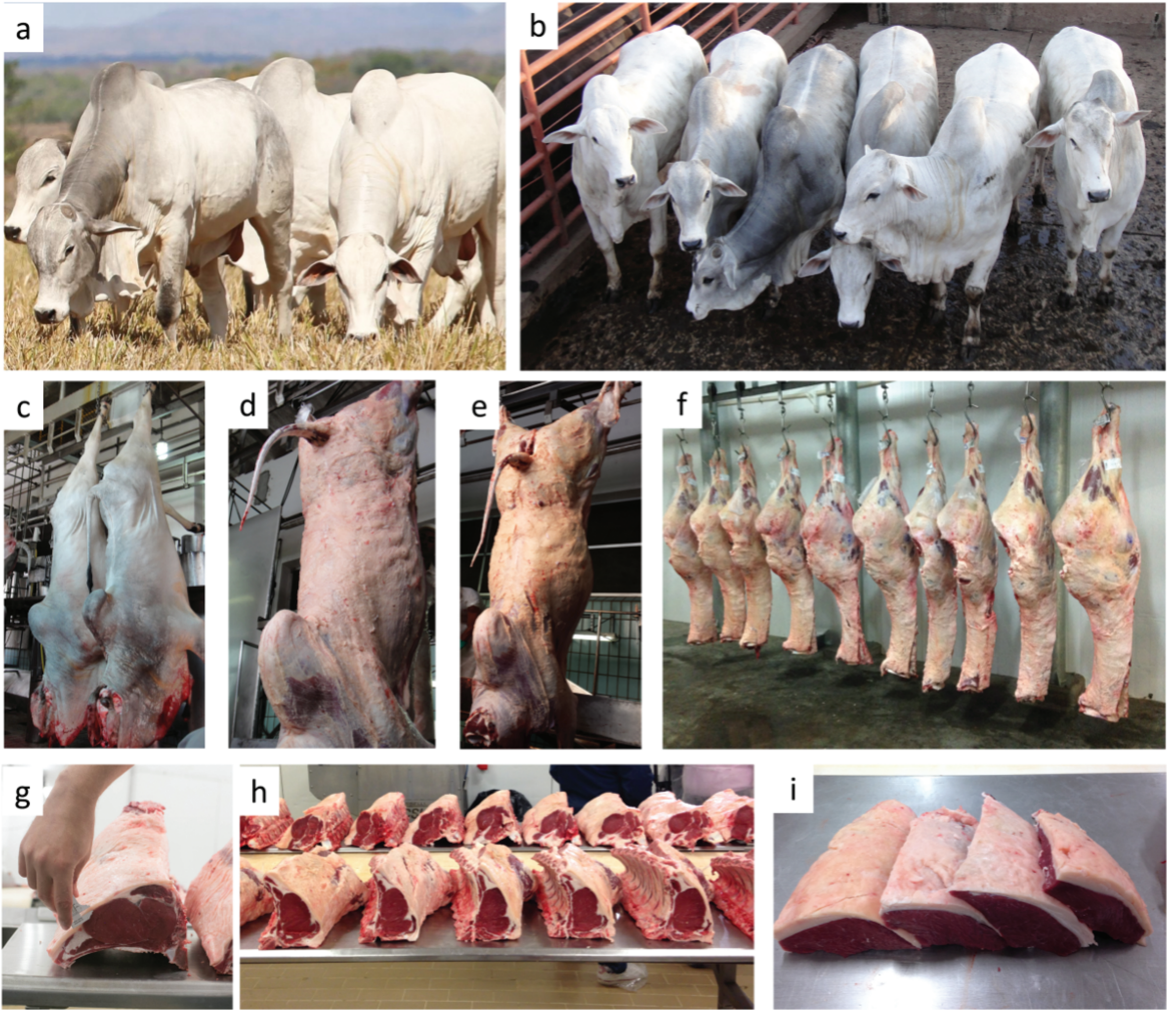
Examples of Nellore cattle, carcass, and beef cuts in Brazil. (a) Nellore sires; (b) Nellore young bulls at slaughterhouse lairage; (c) Nellore carcasses after slaughter; (d-e) Nellore hot carcasses; (f) Nellore cold hindquarter; (g) Measuring rib-eye fat thickness; (h) Nellore beef rib; (i) Picanha/Culotte/Rump cap cut of Nellore carcass. (Photos by Sérgio Pflanzer).

Nellore (*Bos taurus indicus*) is the main beef cattle breed produced in Brazil (Figure 1), which was introduced from India during the 19th century. The establishment of the Nellore breed in Brazil can be primarily attributed to its heat resistance, ability to thrive on poor-quality forages, and easy calving. Currently, more than 80% of Brazil’s beef cattle population comprises purebred or hybrids from the Nellore breed (Bonin et al., 2021). The adoption of crossbred animals, involving *Bos indicus* cows with European or composite breeds, has already become a reality within the Brazilian beef industry. A survey conducted by Silvestre and Millen, (2021) with 36 feedlot cattle nutritionists reported that 52% of their clients were feeding crossbred animals.

## Beef Market Statistics

The animal protein production sector has faced many challenges during the pandemic. The global cessation in 2019 due to the Covid-19 pandemic and its extension into 2021 required the entire Brazilian meat production sector to undertake extensive work to reinforce sanitary controls and guarantee the quality and safety of the Brazilian meat to the consumer’s table. Fortunately, an examination of the meat sector statistics reveals that the industry has successfully maintained its pivotal role in the globe as evidenced by record export revenues.

Brazilian beef exports have demonstrated consistent growth since the beginning of the pandemic. As reported by ABIEC, (2022), the Brazilian beef herd numbered approximately 196.47 million heads in 2021, with a corresponding slaughter count of 39.14 million heads. The volume of meat produced reached 9.71 million tons in terms of beef carcass-weight equivalent and exported close to 2.48 million metric tons. Among the beef cattle slaughtered in 2021, 17.19% originated from feedlot systems, while 82.81% were from grazing systems. The top four destinations for Brazilian *in natura* beef exports in 2022 were China, Hong Kong, Chile, and the USA, representing more than 90% of the total exported beef volume (ABIEC, 2022). In 2023, China temporarily imposed an embargo on Brazilian meat exports due to an atypical and isolated case of Bovine Spongiform Encephalopathy, which generated speculation about market growth. However, China rescinded the temporary embargo on Brazilian meat exports after both nations agreed upon sanitary protocols.

The total revenue estimated from the Brazilian beef cattle slaughtered was estimated at 35,705.1 million US dollars (ABIEC, 2022). However, the Brazilian per capita beef consumption was estimated at 34.37 kg/year, which represents a decrease of 7.75 kg/year compared to the 2018 estimation (ABIEC, 2019). Despite Brazil achieving a record in beef export volume, a reduction in domestic beef consumption has been evident since the Covid-19 pandemic started. The increase in prices and a subsequent reduction in the purchasing power of Brazilian consumers shifted beef consumption preferences towards chicken and pork products.

Brazil is the largest beef supplier in the world and has played an important role in food safety, guaranteeing quality, and sanitary standards. However, there is still room for improvements especially related to meat quality standards and sustainability of beef production systems.

## Brazilian System of Beef Carcass Classification and Certifications

In 2002, the Brazilian System of Identification and Certification of Bovine and Buffalo Origin (SISBOV) was implemented to meet the needs of European Union’s demand for certification requirements, being the main importer of Brazilian meat at the time. Even though traceability certification is a requirement of some Brazilian beef export destinations, the participation of the producers in the traceability system remains voluntary (Andrade et al., 2020).

Brazil does not have a standardized national program for beef quality and yield grading. In 2004, the Ministry of Agriculture, Livestock, and Food Supply (MAPA), which is the federal agency dedicated to promoting agribusiness development, approved a Normative Instruction No. 9 (NI.9) establishing the Brazilian Bovine Carcass Classification System (Table 1). However, individual slaughterhouse companies are allowed to employ distinct metrics for carcass grading to meet market/consumer requirements (Silvestre and Millen, 2021). Also, there are few private sector companies that conduct beef carcass quality grading based on USDA or AUS-MEAT standards to provide grading criteria for specific market brands sold within Brazil.

**Table 1.** Current Normative Instruction for cattle carcass classification in Brazil

Characteristics	Classification
Age (dentition)	zero, two, four, six, or eight permanent incisors
Gender	Female, castrated male, uncastrated male, and cull cow
Carcass weight	Hot carcass weight (kg)
Fat coverage	Absent (<1 mm), slight (1 to 3 mm), medium (4 to 6 mm), uniform (7 to 10mm), and excessive (>10 mm)

Source: Ministry of Agriculture, Livestock, and Food Supply.

While NI No. 9/2004 is currently in effect, some Brazilian industries export meat to the European Union through the BRASIL classification system, as stipulated by federal regulation No. 612/1989. This regulation was implemented to fulfill the Hilton Quota demands, which requires carcasses from young steers and heifers with a maximum of four permanent incisors, and exclusively raised on pastures (Pardi et al., 1996). In the BRASIL system, hot carcasses are placed into classification types based on sex, weight, age, conformation, and fat coverage parameters (Table 2), without any assessment of eating quality attributes. However, a slight change has been made to meet Hilton Quota requirements. According to regulation No. 72/2020, which regulates export quotas, beef carcasses classified as “B” with slight or medium fat coverage are eligible for the Hilton Quota.

**Table 2.** Characteristics of carcass grading according to the No 612/1989 regulation

	Sex/weight	Age	Carcass conformation^1^	Fat coverage
**B**	Female (>180 kg), castrated (>210 kg), and uncastrated male (>210 kg)	Uncastrated male: 0 permanent incisors Female and castrated male: ≤ 4 permanent incisors	Convex, subconvex, and rectilinear	Slight (1–3 mm), medium (4–6 mm), and uniform^2^ (7–10 mm)
**R**	Female (>180 kg), and castrated male (>210 kg)	4–6 permanent incisors	Convex, subconvex, rectilinear, and sub rectilinear	Uniform (7–10 mm) and excessive (>10 mm)
**A**	Female (>180 kg), castrated (>210 kg), and uncastrated male (>210 kg)	Uncastrated male: 0 permanent incisors Female and castrated male: 4–6 permanent incisors	Convex, subconvex, rectilinear, and sub rectilinear	Absent (<1 mm) and excessive (>10 mm)
**S**	Female (>180 kg), and castrated male (>225 kg)	Eight permanent incisors	Convex, subconvex, rectilinear, and sub rectilinear	No specification
**I**	Female (<180 kg), castrated (<225 kg), and uncastrated male	Eight permanent incisors	Convex, subconvex, rectilinear, and sub rectilinear	No specification
**L**	No specification	No specification	Concave	No specification

^1^Carcass conformation are classified from best to worst as follows: convex, subconvex, rectilinear, sub-rectilinear, and concave. Convex carcasses are characterized by having superior muscularity and rounded shape in the round portion, while concave carcasses are considered the least desirable, exhibiting lower muscular development.

^2^Uniform fat coverage was removed from the Hilton Quota requirements.

Carcass conformation classification is based on muscular development of the round portion of the hindquarter, ranked from best to worst as follows: convex, subconvex, rectilinear, sub-rectilinear, and concave. Convex carcasses have the best conformation due to superior muscularity and rounded shape, while concave carcasses are considered the least desirable, exhibiting lower muscular development. Carcasses with better conformation tend to have a lower proportion of bone and a higher proportion of edible content. Also, greater muscle hypertrophy provides better-looking cuts for the most demanding consumers.

The BRASIL system is not efficient in accurately categorizing carcasses of similar types within the same group. For instance, carcasses of three sexual classes (female, uncastrated, and castrated male) combined with three different fat coverage scores (slight, medium, and uniform) and three carcass conformation types (convex, subconvex, and rectilinear) are placed within the same group type “B”. This categorization can lead to a large variability of carcass characteristics, resulting in a lack of uniformity in meat quality parameters and, consequently, in boneless lean meat yield.

Furthermore, producers can also adhere to specific bonus programs such as the “Novilho Precoce MS.” This program is established in Mato Grosso do Sul state and provides tax benefits for producers who harvest beef animals with four permanent teeth maximum, fat deposition between 1 and 10 mm, carcass weight > 225 kg for steers and bulls, and > 180 kg for heifers (Amaral et al., 2020). Carcasses that do not fit into the type “B” classification for export within the Hilton Quota and for a specific certification program usually are not classified for the domestic market.

Through a national survey involving nutrition consultants (*n* = 23), feedlot owners (*n* = 21), and packer-owned feedlots (*n* = 8), Andrade et al., (2020) identified that fat cover and weight were the factors most cited in determining carcass discounts or bonuses. In addition, characteristics such as steer (2–3 years), heifers, young bulls, age or maturity (through carcass dentition), and whether the animal is Angus or Hereford were indicated as factors determining carcass bonuses. Ribeye area, marbling, meat cut weight, meat tenderness, meat flavor, meat color, and fatty acid profile were characteristics less cited as factors for gaining carcass bonuses. Intriguingly, the feedback from respondents indicated that characteristics contributing to meat quality and boning yields hold relatively low significance in terms of increasing the average compensation extended to producers.

## Private Carcass Grading System in Brazil

Private initiatives targeting consumer satisfaction of beef products such as attributing different standards to meat cuts are becoming more common. According to Felício, (2016), meat cuts are classified as Extra Premium, Premium, Grill Line, and Standard. Extra Premium is characterized by *Bos taurus taurus* breeds with ages up to 2 years old and an average carcass weight of 300 kg. The amount of Extra Premium cuts produced is not enough to meet domestic market demand. Thus, Brazil imports special cuts from Uruguay, Argentina, and Australia. Cuts resulting from crossbred animals (*Bos taurus taurus* × *Bos taurus indicus*) are usually classified as Premium due to less variation in meat quality parameters such as tenderness, flavor, and juiciness. Grill cuts are categorized based on slaughterhouses’ criteria of age and fat coverage classification. In general, carcasses of young animals with a medium or uniform fat coverage are selected to meet the requirements of the slaughterhouses’ own brands. Grill cuts reach the retail with at least 15 days of aging after packaging. Lastly, Standard cuts are obtained from older animals such as bulls, or cuts categorized with absent or slight fat coverage.

## Brazilian Beef Quality and Consumers

Understanding the preferences of Brazilian beef consumers is important in planning strategies to both attract and retain customers. Boito et al., (2021) evaluated Brazilian and Spanish perceptions regarding beef quality, particularly, investigating intrinsic and extrinsic attributes of beef that impact purchase decisions and subsequent repeat purchases. Their study revealed that Brazilian and Spanish consumers are primarily concerned with extrinsic characteristics of the meat, such as feeding system, age, slaughter date and location, origin, and animal sex. In contrast, intrinsic attributes of the product are evaluated based on personal qualitative preferences and ethical principles, guiding the selection of a quality product. Then Brazilian consumers prioritize the general appearance of the meat and cooking time, while Spanish consumers prioritize tenderness, juiciness, and flavor. This could be explained due to the lack of standards in Brazilian meat compared to Spain (Boito et al., 2021).

The process of meal preparation in Brazilian and other South American households, particularly during weekends and holidays, diverges significantly from many other regions due to the cultural tradition of treating slow-cooked grilled meat as an event in itself (Carvalho et al., 2014). In fact, Brazilian consumers expect affordable meat products with high organoleptic, sanitary, and nutritional quality and produced under high ethical standards (Hötzel and Vandresen, 2022).

## Recommendations for a New Brazilian Beef Carcass and Grading System

The establishment of an official carcass grading system in Brazil must be persuaded. By classifying and grading carcasses and meat, we not only facilitate communication in the cattle and carcass market industry but also generate valuable information for our productive sector. This information enables us to make more informed and precise decisions, with the goal of optimizing both the productivity and quality indicators of our national beef.

In this context, as a first step to implementing a Brazilian beef carcass and grading system, the authors suggest a system based on two phases. The first phase would involve the classification of hot carcasses based on weight, sex, age, and distribution of fat cover, without the need to combine those criteria. This would provide a solid foundation for negotiations between packers and producers. The second phase would focus on grading the carcasses by combining the previous indicators. In this stage, the previously evaluated indicators would be combined to form different categories, namely “Optimal,” “Selected,” and “Common”, for example. Optimal carcasses would be those from young animals with excellent fat cover. Selected carcasses would include those from animals of intermediate age or with limited fat cover. The remaining carcasses would be classified as Common.

When it comes to Optimal carcasses, which have the potential for intramuscular fat deposition, they could be graded as “Excellent” if the level of assessed intramuscular fat in the cold carcasses equals or exceeds the moderate level (according to the USDA system, for instance). Also, adding ultimate pH and meat color as grading parameters is essential to achieve high-quality products. This two-phase classification and grading system would incentivize producers to deliver a more uniform carcass in terms of quality to the packers. This, in turn, would enable retailers to establish pricing based on quality, and consumers would access a more standardized range of products in the market.

Also, we believe that the incorporation of technological equipment and artificial intelligence systems is crucial to ensure efficiency in the classification and grading process. The utilization of objective and noninvasive technologies within the industry not only facilitates carcass evaluation and data processing but also speeds up decision-making.

## Conclusion

Carcass classification in Brazil is based primarily on age, gender, weight, and fat coverage and there is no national grading system. Therefore, the slaughterhouse plants apply individual grading systems based on their export market demands. Currently, there is a desire to establish a new Brazilian classification and grading system of bovine carcasses, including eating quality parameters such as pH, marbling score, and meat color. However, our challenge is to establish a system that covers the entire Brazilian territory, which is characterized by different profiles of production systems, types of climates, and breeds.

Acknowledgement

This manuscript was invited for submission by the American Meat Science Association. The views expressed in this publication are those of the author(s) and do not necessarily reflect the views or policies of the American Meat Science Association, the journal, or the publisher.

## About the Authors

**Cris Nunes** is an Animal Scientist and Postdoctoral Researcher within the Department of Animal Science at the Federal University of Viçosa (UFV), Brazil. She earned her B.S. from the Universidade Estadual de Mato Grosso do Sul (UEMS), Brazil, which was followed by a M.S. from the Universidade Federal de Viçosa (UFV), Brazil. Dr. Nunes’ research focuses on technologies to improve meat quality standards, including assessment of chilling rates’ impact on beef quality, utilization of DEXA technology to predict fat content, and genomic breed traceability to ensure transparency to the consumers and protect the identity and value of the product. In 2022, as a Visiting Scholar at Washington State University (WSU), Dr. Nunes developed part of her Ph.D. in learning new approaches to improve livestock sustainability. **Corresponding author:**cris.nunes@ufv.br

**Sérgio Pflanzer** earned his bachelor’s degree in veterinary medicine from Pontifícia Universidade Católica do Paraná (PUC-PR) in 2005. Afterward, Dr. Pflanzer received the titles of Master and Doctor in Food Technology from the Faculty of Food Engineering from UNICAMP in 2008 and 2012, respectively. Currently, he holds a professor position at the Faculty of Food Engineering, UNICAMP, Brazil, teaching within the areas of expertise on Food Hygiene and Meat Characteristics and Processing. Furthermore, Dr. Pflanzer’s research focuses on pre- and post-slaughter factors affecting the meat quality of different species, with a particular emphasis on beef.

**Jonatã H. Rezende-de-Souza** is a Food Scientist from the Federal University of Mato Grosso, Brazil. He holds a Master’s degree and is currently a Ph.D. student in Food Technology at the Faculty of Food Engineering, University of Campinas, Brazil. He works with beef quality research, focusing on the aging process and the genetic influence on the final quality of meat. Additionally, his research extends to seafood, particularly in adding value to native fish species through quality assessment and the development of new products.

**Mario Chizzotti** is currently an Assistant Professor and holds the position of Dean of the Agricultural Sciences at the Federal University of Viçosa (UFV). He earned his M.S. (2004) and Ph.D. (2007) degrees in Animal Science from UFV. His teaching areas focus on the meat production chain, with emphasis on Meat Quality, Growth Physiology, and Precision Livestock Farming, while his research focuses on Bioenergetics and Nutritional Requirements, Artificial Intelligence and Modeling in Animal Production, Growth Physiology, and Meat Science. Dr. Chizzotti’s research program has been supported by many funding agencies, including the Fundação de Amparo à Pesquisa de Minas Gerais (FAPEMIG), Conselho Nacional de Desenvolvimento Científico e Tecnológico (CNPq), Coordenação de Aperfeiçoamento de Pessoal de Nível Superior (CAPES), Instituto Nacional de Ciência e Tecnologia de Ciência Animal (INCT-CA), and animal nutrition private companies.

## References

[CIT0001] ABIEC. 2019. Beef report perfil da pecuária no Brasil. [accessed August 20, 2023]. https://abiec.com.br/wp-content/uploads/sumario2019portugues.pdf

[CIT0002] ABIEC. 2022. Beef report overview of livestock in Brazil. [accessed August 20, 2023]. https://www.abiec.com.br/en/publicacoes/beef-report-2022-2/

[CIT0003] Amaral TB , Fernandes AFA, Rosa GJ. 2020. PSXI-5 Best production practices for improvement of Beef Cattle carcass quality. J. Anim. Sci. 98(4):384. 10.1093/jas/skaa278.675

[CIT0004] Andrade TS , Albertini TZ, Barioni LG, de Medeiros SR, Millen DD, Dos Santos ACR, Goulart RS, Lanna DPD. 2020. Perception of consultants, feedlot owners, and packers regarding management and marketing decisions on feedlots: A national survey in Brazil (Part II). Can. J. Anim. Sci. 100(4):759–770. 10.1139/cjas-2019-0220

[CIT0005] Boito B , Lisbinski E, Campo MDM, Guerrero A, Resconi V, de Oliveira TE, Barcellos JOJ. 2021. Perception of beef quality for Spanish and Brazilian consumers. Meat Sci. 172:108312. 10.1016/j.meatsci.2020.108312 33011632

[CIT0006] Bonin MN , Pedrosa VB, da Luz e Silva S, Bünger L, Ross D, da Costa Gomes R, de Almeida Santana MH, Córdova Cucco D. de, de Rezende FM, Ítavo LCV, et al. 2021. Genetic parameters associated with meat quality of Nellore cattle at different anatomical points of longissimus: Brazilian standards. Meat Sci. 171:108281. 10.1016/j.meatsci.2020.108281 32892086

[CIT0007] Carvalho AM , César CLG, Fisberg RM, Marchioni DM. 2014. Meat consumption in Sao Paulo – Brazil: Trend in the last decade. PLoS One 9(5):Article e96667. 10.1371/journal.pone.0096667 24792240 PMC4008596

[CIT0008] Chiari L , Gomes RC, Bungenstab DJ, Egito AA, Catto JB, Araujo FR, Alves FV, Almeida RG, Barbosa RA. 2021. Pecuária de corte otimização do uso da terra e adoção da intensificação sustentável. In: Telhado SFP., Capdeville G, editors. Tecnologias poupa-terra 2021. Brasília, DF: Embrapa; p. 141–156.

[CIT0009] Felício PE. 2016. Brasil: apreciadores de carne bovina já encontram boas opções de compra no mercado. Revista Carne Tec. [accessed September 25, 2023]. http://library.carnetec.com.br/publication/?m=28216&i=300408&p=28&ver=html5

[CIT0010] Hötzel MJ , Vandresen B. 2022. Brazilians’ attitudes to meat consumption and production: Present and future challenges to the sustainability of the meat industry. Meat Sci. 192:108893. 10.1016/j.meatsci.2022.108893 35760024

[CIT0011] Lobato JFP , Freitas AK, Devincenzi T, Cardoso LL, Tarouco JU, Vieira RM, Dillenburg DR, Castro I. 2014. Brazilian beef produced on pastures: Sustainable and healthy. Meat Sci. 98(3):336–345. 10.1016/j.meatsci.2014.06.022 25017318

[CIT0012] Malafaia GC , Mores GV, Casagranda YG, Barcellos JOJ, Costa FP. 2021. The Brazilian beef cattle supply chain in the next decades. Livest. Sci. 253:104704. 10.1016/j.livsci.2021.104704

[CIT0013] Pardi MC , Santos IF, Souza ER, Santos JCA. 1996. Epopéia do zebu – um estudo zootécnico-econômico – 1944/1994. Goiania, Go: Editora UFG; p. 126

[CIT0014] Rosado Júnior AG , Lobato JFP. 2010. Implementation of a performance indicators system in a beef cattle company. Revista Brasileira de Zootecnia 39(6):1372–1380. 10.1590/s1516-35982010000600029

[CIT0015] Silvestre AM , Millen DD. 2021. The 2019 Brazilian survey on nutritional practices provided by feedlot cattle consulting nutritionists. Revista Brasileira de Zootecnia 50:1–25. 10.37496/RBZ5020200189

[CIT0016] USDA. 2023. Livestock and poultry: World markets and trade. [accessed August 20, 2023]. https://apps.fas.usda.gov/psdonline/circulars/livestock_poultry.pdf

